# Nε-Carboxymethyl-Lysine Modification of Extracellular Matrix Proteins Augments Fibroblast Activation

**DOI:** 10.3390/ijms242115811

**Published:** 2023-10-31

**Authors:** Harshavardhana H. Ediga, Patrick Hester, Adithi Yepuri, Geereddy Bhanuprakash Reddy, Satish K. Madala

**Affiliations:** 1 Division of Pulmonary, Critical Care and Sleep Medicine, Department of Internal Medicine, University of Cincinnati, Cincinnati, OH 45267-0564, USAhesterps@mail.uc.edu (P.H.);; 2 Department of Biochemistry, ICMR-National Institute of Nutrition, Hyderabad 500007, India; reddyg.bp@icmr.gov.in

**Keywords:** advanced glycation end products, Nε-carboxymethyl-lysine, extracellular matrix, myofibroblast transformation, idiopathic pulmonary fibrosis

## Abstract

The extracellular matrix (ECM) is a dynamic complex protein network that provides structural integrity and plays an active role in shaping fibroblast behavior both in health and disease. Despite its essential functions, the impact of age-associated post-translational modifications on ECM-driven fibroblast activities such as proliferation, survival, fibroblast-to-myofibroblast transformation (FMT), and extracellular matrix production remains largely unknown. Nε-carboxymethyl-lysine (CML) is one of the well-characterized advanced glycation end-products (AGEs) that can occur on lysine residues within ECM proteins through non-enzymatic glycation. In this study, we determined the accumulation and the effects of the CML-modified ECM (CML-ECM) on fibroblast activation. Immunostainings and immunoblot analysis demonstrated significant increases in CML-AGE content in idiopathic pulmonary fibrosis (IPF) compared to age-matched healthy lungs. Gene expression analysis and fibroblast activation assays collectively implicate the ECM as a negative regulator of fibroblast activation. Notably, the CML modification of the ECM resulted in a significant decrease in its anti-fibrotic effects including proliferation, FMT, apoptosis, and ECM production. Together, the results of this study revealed an unexplored pathological role played by the CML-ECM on fibroblast activation, which has wide implications in IPF and other fibrotic diseases.

## 1. Introduction

Idiopathic pulmonary fibrosis (IPF) is a fatal fibrotic lung disease that is associated with fibroblast activation and excessive deposition of the extracellular matrix (ECM) in the lung parenchyma, and its incidence rate steadily increases with aging. The median survival time after diagnosis is typically estimated to be around 3 to 5 years [1,2,3,4]. The exact cause of IPF is not fully understood, but it is believed to result from a combination of genetic predisposition, environmental factors, and abnormal wound-healing responses [1,5,6]. The pathogenic changes in IPF are characterized by fibroblast dysfunction and aberrant fibroblast–ECM signaling that lead to excessive proliferation, myofibroblast transformation (FMT), and impaired apoptotic clearance of myofibroblasts [7,8]. This results in an excessive deposition of the ECM and the formation of fibrotic scars that impair lung function and gas exchange [9].

The ECM is a complex network of proteins and proteoglycans that provide structural support and contribute to cell–matrix interactions underlying the homeostasis of the lung. The cells and the ECM share a dynamic and reciprocal relationship wherein cells are involved in the production and remodeling of the ECM, which in turn mediates the composition and topography of the ECM. Conversely, the ECM exerts an influence on cell behaviors and functions [10]. In IPF, ECM proteins such as collagens, elastin, fibronectin are overproduced, leading to a stiffer and less flexible ECM [9,11]. This altered ECM composition can impair cellular functions associated with interactions with the ECM and lung function, reduce tissue elasticity, and contribute to the development of age-related fibrosis [12]. Also, aging and lung injury alter the composition and structure of the ECM due to post-translational modifications (PTMs) involving chemical modifications on proteins, which can impact and disrupt cell–matrix interactions in IPF [13,14,15]. Glycation, nitrosylation, oxidation, and the cross-linking of ECM proteins by PTM have been shown to result in impaired tissue elasticity and a more rigid ECM, thereby serving as drivers of pathology in various age-related diseases, including IPF [16].

Non-enzymatic glycation is a process wherein a carbonyl (aldehyde or keto) group of sugar molecules reacts covalently with amino groups on proteins and upon a series of subsequent reactions/rearrangements leads to the formation of more stable and irreversible advanced glycation end-products (AGEs) without the involvement of enzymes [17,18,19]. Although AGEs comprise a large number of heterogeneous chemical adducts, Nε-carboxymethyl-lysine (CML) is one of the well-characterized AGEs and most prevalent AGEs in vivo [20]. Although the formation of AGEs including CML occurs during normal metabolic processes albeit at a lower rate, their accumulation increases with aging and more so in clinical conditions such as diabetes due to accelerated formation [21,22]. Studies have shown that people with diabetes may experience AGE accumulation in the lungs and decreased lung function parameters, such as forced expiratory volume in one second and forced vital capacity, which are indicators of lung capacity and airflow [23,24]. 

Biochemically, the formation of AGEs will exacerbate oxidative damage and inflammation, which leads to mechanical tissue stiffness and may in turn help in the disease progression [25,26]. The mechanisms whereby injury, aging, and hyperglycemia-driven modification alter homeostasis between the ECM and fibroblast interactions that exacerbate the severity of fibrotic lung disease remain to be defined. Recent published studies on the histological evaluation of IPF tissue suggest extensive post-translational modifications of the ECM including AGE formation in mature fibrotic lesions of IPF [14,15]. In pulmonary fibrosis, there is often chronic inflammation and tissue damage that can augment non-enzymatic glycation and CML formation [15]. In support, lysine residues in proteins within the ECM can become glycated and accumulate in tissues with limited turnover. In particular, the published studies suggest measuring CML levels can be used as a marker of tissue damage and fibrosis [15,27,28]. Although excessive glycation and the formation of AGEs on long-lived proteins such as the ECM can alter their structure and function [29,30], their accumulation and contribution to fibroblast activation and pulmonary fibrosis in IPF remained unexplored. Furthermore, the role of specific types of AGEs that accumulate and contribute to the ECM and fibroblast interactions in IPF or other fibrotic lung diseases is unknown.

Here, we aim to assess the accumulation of the CML-modified ECM (CML-ECM) in IPF lungs and investigate the fibroproliferative effects of the CML-ECM using fibroblast activation assays. To achieve this goal, we isolated a decellularized ECM from mouse and human lungs and subjected them to CML modification. We show that the ECM inhibits proliferation and ECM production and also induces apoptosis in fibroblasts. In contrast, CML modification of the ECM is sufficient to impair its anti-fibrotic effects on fibroblasts including proliferation, transformation to myofibroblasts, and apoptosis, revealing a role for CML-AGEs in fibrotic lung diseases.

## 2. Results

### 2.1. Accumulation of CML-Modified ECM in Fibrotic Lesions of IPF Lungs

**Figure 1:**
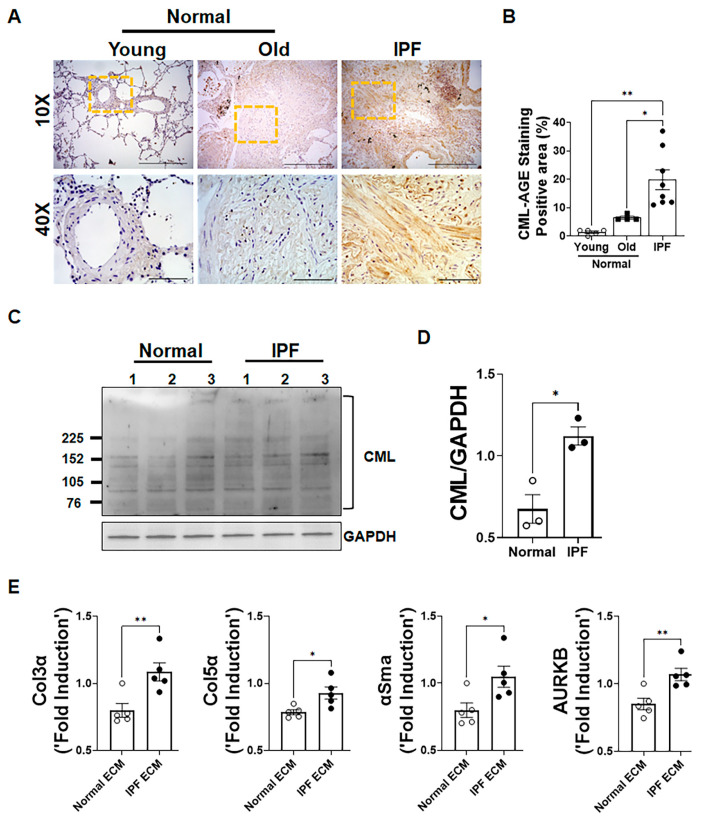
Accumulation of CML-modified extracellular matrix (ECM) proteins in fibrotic lesions of IPF lungs and its effect on expression of fibrosis-related genes. (**A**) Representative immunostained images of normal young, normal old, and IPF lungs against carboxymethyllysine (CML) were captured at 10× (scale bar, 500 µm) and 40× (scale bar, 100 µm) magnification. (**B**) Quantification of CML immunostaining in young, old, and IPF lung sections using the BZ-X analyzer. One-way ANOVA was used (* *p* < 0.05, ** *p* < 0.01; n = 4–8/group). (**C**) Immunoblotting of lung lysates from normal and IPF patients with anti-CML and anti-GAPDH antibodies. (**D**) Normalized CML-adduct levels relative to GAPDH. Student’s two-tailed *t*-test was used (* *p* < 0.5; n = 3/group). (**E**) Quantification of gene transcripts (Col3α, Col5α, αSma, and Aurora kinase B) in healthy lung resident fibroblasts treated with a decellularized ECM from the lungs of healthy individuals and IPF patients. Treatment involved an ECM (1 µg/mL) in DMEM media with 1% FCS for 16 h. Student’s two-tailed *t*-test was used (* *p* < 0.05, ** *p* < 0.01; n = 5/group).

To determine the accumulation of CML modifications in healthy (young and old) humans and IPF patient lungs, we performed immunohistochemistry on lung sections with antibodies specific to CML-AGE adducts. We were unable to detect CML-AGEs in human young healthy lung sections, while there was a detectable staining of CML-AGEs in human healthy old lungs. However, there was a greater CML-AGE staining in mature fibrotic lesions of human IPF lungs compared to normal lung sections (Figure 1A). We also quantified the percent CML-AGE staining from healthy and IPF lung sections. A significant increase in CML-AGE staining was observed in lung sections of IPF individuals compared to healthy controls (Figure 1B). To further confirm CML accumulation in IPF lungs, we also performed immunoblot analysis using antibodies specific to CML-AGE in total lung lysates from normal and IPF (Figure 1C). We observed a significant increase in CML adducts formation on proteins from IPF lung lysates compared to healthy controls (Figure 1D). To determine the effects of a decellularized ECM on the expression of fibrosis-associated genes, we treated fibroblasts with decellularized ECM extract from IPF and healthy lungs and measured the expression of ECM genes. As shown in Figure 1E, we observed a significant increase in the expression of ECM gene transcripts collagen 3α, collagen 5α, αSma and AurkB in fibroblast treated with the ECM from IPF compared to an ECM from healthy age-matched decellularized lungs.

### 2.2. CML Modification Attenuates ECM-Dependent Inhibition of Collagen and FN1 Gene Expression in Fibroblasts

**Figure 2:**
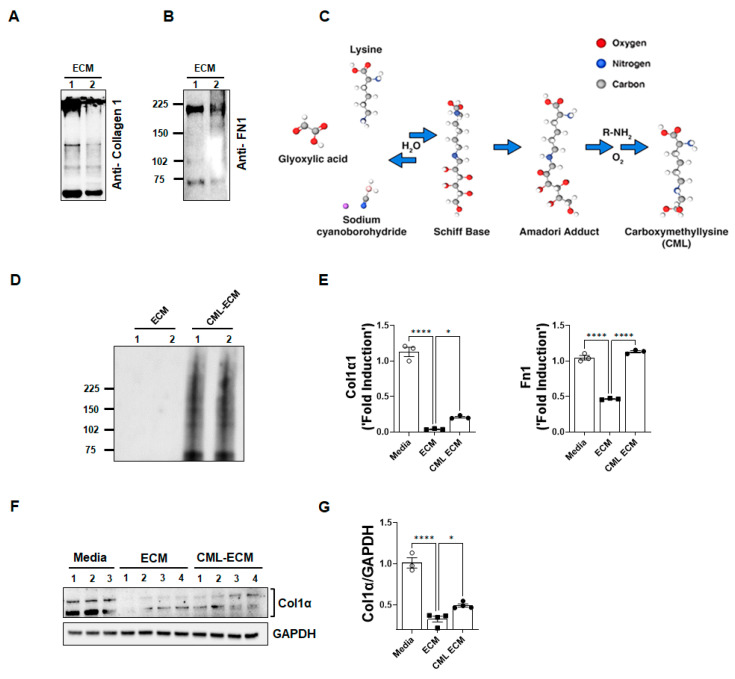
CML modification attenuates ECM-dependent inhibition of collagen and FN1 gene expression in fibroblasts. (**A**) Soluble ECM was extracted from decellularized mouse lungs, and we performed immunoblot analysis for the presence of collagen1. (**B**) Soluble ECM was extracted from decellularized mouse lungs, and we performed an immunoblot analysis for the presence of fibronectin1. (**C**) Schemata showing the formation of CML in proteins through a Schiff base intermediate upon reaction between the glyoxylic acid and protein in the presence of sodium cyanoborohydride, which then undergoes the Amadori rearrangement to form an irreversible AGE adduct called CML. (**D**) Immunoblot analysis using anti-CML antibodies shows CML-AGE formation in the ECM when incubated with glyoxylic acid and sodium cyanoborohydride. (**E**) Quantification of gene transcripts (Col1α1 and Fn1) in lung resident fibroblasts treated with an ECM and CML-ECM at 5 µg/mL compared to media-treated fibroblasts for 16 h. One-way ANOVA was used (* *p* < 0.05, **** *p* < 0.0001; n = 3/group). (**F**) Immunoblotting for Col1α1 and GAPDH in lung fibroblasts treated with media, ECM, or CML-ECM for 72 h. (**G**) Quantification Col1α1 levels normalized to GAPDH. One-way ANOVA was used (* *p* < 0.05, **** *p* < 0.0001; n = 3–4/group).

To assess the effects of the ECM, we prepared a soluble ECM extract from the decellularized lungs of mice and confirmed the presence of collagen 1 and FN1 in ECM proteins by immunoblotting (Figure 2A,B). To generate a CML-modified ECM via non-enzymatic glycation, a soluble ECM extract was incubated with glyoxylic acid and sodium cyanoborohydride (Figure 2C). The formation of the CML-modified ECM was confirmed by immunoblotting with antibodies against CML adducts (Figure 2D). To determine the effects of the ECM and CML-modified ECM, fibroblasts isolated from mouse lung cultures were treated with media, the ECM, and the CML-ECM. We observed a significant decrease in the expression of collagen 1 and FN1 gene transcripts by the ECM compared to the media control. However, CML modification of the ECM attenuated the inhibitory effects of the ECM on collagen 1 and FN1 gene expression (Figure 2E). Furthermore, we validated the effects of CML modification on ECM-dependent collagen protein production by fibroblasts using immunoblot analysis and observed a significant loss in the ECM-induced inhibition of collagen 1 production by fibroblasts with CML modification (Figure 2F,G).

### 2.3. CML Modification Attenuates ECM-Driven Inhibition of Fibroproliferation

**Figure 3:**
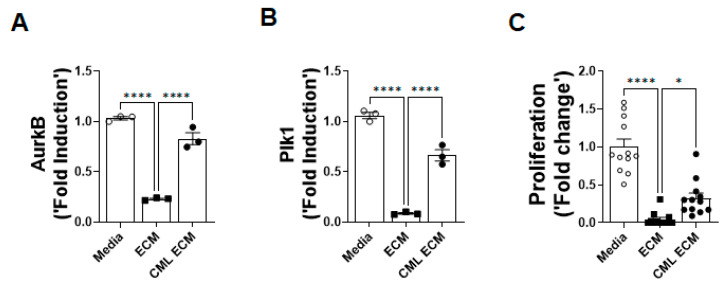
CML modification attenuates ECM-driven inhibition of fibroproliferation. (**A**) Quantification of AurkB transcripts using RT-PCR in lung resident fibroblasts treated with media, the ECM, or the CML-ECM (5 µg/mL) for 16 h. One-way ANOVA was used (**** *p* < 0.0001; n = 3/group). (**B**) Quantification of Plk1 transcripts using RT-PCR in lung resident fibroblasts treated with media, the ECM, or the CML-ECM (5 µg/mL) for 16 h. One-way ANOVA was used (**** *p* < 0.0001; n = 3/group). (**C**) Percent BrdU incorporation in fibroblasts treated with media, an ECM, or a CML-ECM (5 µg/mL) for 48 h. One-way ANOVA was used (* *p* < 0.05, **** *p* < 0.0001; n = 12/group).

To determine the effects of CML modification on ECM-induced fibroproliferation, lung resident fibroblasts isolated from mouse lung cultures were treated with media, the ECM, and the CML-ECM. We assessed quantitative changes in mitotic gene expression and observed a significant decrease in the expression of fibroproliferative genes, Aurora kinase B (AurkB) and Polo like kinase 1 (Plk1), in fibroblasts treated with the ECM compared to those treated with media. This decrease in the expression of AurkB and Plk1 by the ECM was significantly attenuated with CML modification (Figure 3A,B). To validate these transcriptional changes in relation to fibroproliferation, we performed BrdU incorporation-based proliferation assays and observed that CML modification attenuated the anti-proliferative effects of the ECM on fibroblasts (Figure 3C).

### 2.4. CML Modification of ECM Attenuates Fibroblast to Myofibroblast Transformation

**Figure 4:**
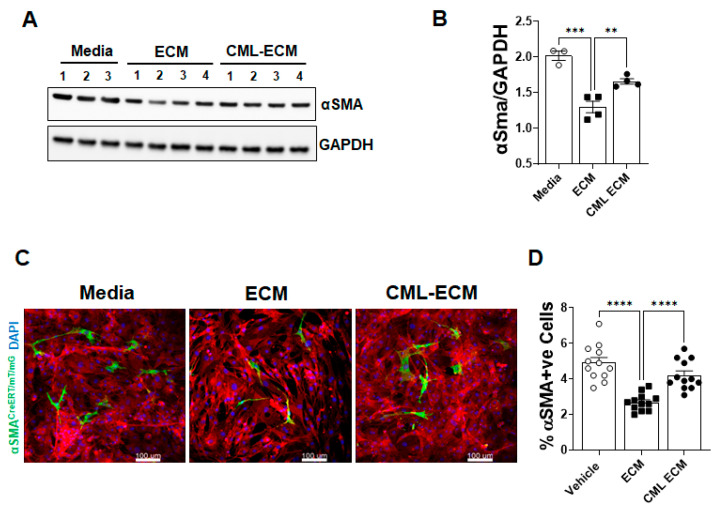
CML modification of ECM attenuates fibroblast-to-myofibroblast transformation (FMT). (**A**) Immunoblotting for αSma and GAPDH in fibroblasts treated with media, ECM, and CML-ECM (5 µg/mL) for 48 h. (**B**) αSma protein levels were normalized to GAPDH in fibroblast lysates. One-way ANOVA was used (** *p* < 0.01, *** *p* < 0.001; n = 3–4/group). (**C**) Lung fibroblasts from myofibroblast reporter mice were treated with media, ECM, and CML-ECM (5 µg/mL) in the presence of 4-hydroxytamoxifen for 72 h. Representative images at 10× magnification. Scale bars, 100 µm. (**D**) The percent of myofibroblasts in the total lung fibroblasts treated with media, ECM, or CML-ECM for 72 h. One-way ANOVA was used (**** *p* < 0.0001, n = 12/group).

To test effect of the ECM and CML-ECM on myofibroblast transformation, we quantified the levels of αSma protein in lung resident fibroblasts treated with media, the ECM, or the CML-ECM. We observed a significant reduction in αSma in fibroblasts treated with the ECM compared to media, and this decrease was significantly attenuated with CML modification of the ECM (Figure 4A,B). To determine the effects of the ECM and CML-ECM on FMT, we used a cell fate-mapping strategy based on the lineage-specific expression of αSMA in lung-resident fibroblasts isolated from αSMA reporter mice [31]. In this model, the activation of Cre recombinase under the control of the αSMA promoter removes the STOP cassette between the 2 loxP sites, which irreversibly changes the expression of membrane tomato (mT) to membrane GFP (mG) in αSMA-expressing lung-resident fibroblasts. Lung-resident fibroblasts isolated from αSMA reporter mice were treated with media, the ECM, or the CML-ECM in the presence of 4-hydroxy-tamoxifen for 72 h. We observed a significant decrease in the number of myofibroblasts (green cells) in reporter fibroblasts treated with the ECM compared to media. This decrease in FMT was attenuated with the CML modification of the ECM, which supports increases in the percent of myofibroblasts by the CML-ECM compared to the ECM (Figure 4C,D).

### 2.5. CML Modification of ECM Attenuates Apoptotic Clearance of Fibroblasts

**Figure 5:**
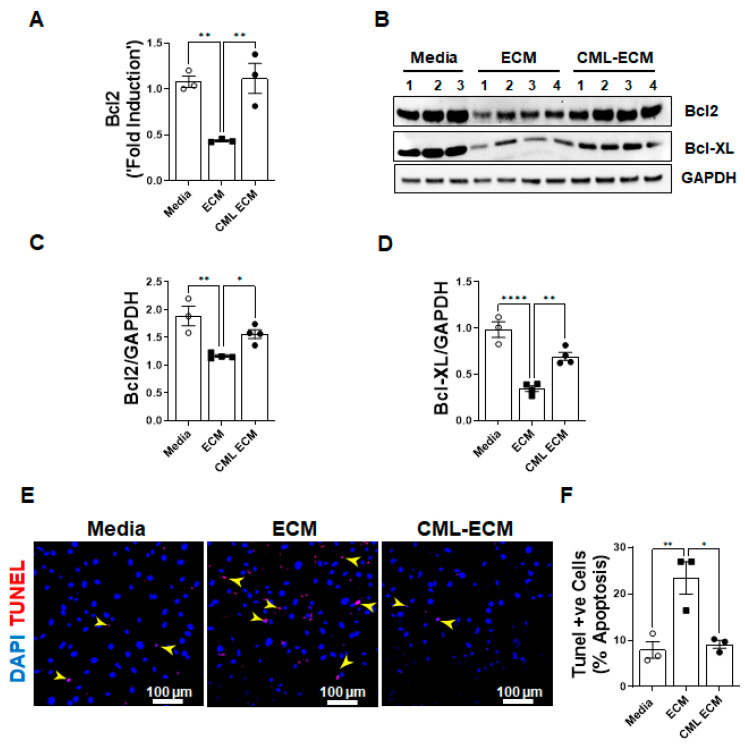
CML modification of ECM attenuates apoptosis in fibroblasts. (**A**) Quantification of Bcl2 gene transcripts in lung resident fibroblasts treated with media, ECM, or CML-ECM (5 µg/mL) for 16 h. One-way ANOVA was used (** *p* < 0.01, n = 3/group). (**B**) Immunoblot analysis of anti-apoptotic proteins (Bcl2 and Bcl-XL) in fibroblasts treated with media, ECM, or CML-ECM (5 µg/mL) for 72 h. (**C**,**D**) Bcl2 and Bcl-XL protein levels were normalized to GAPDH in fibroblast lysates. One-way ANOVA was used (* *p* < 0.05, ** *p* < 0.01, **** *p* < 0.0001; n = 3–4/group). (**E**) Mouse fibroblasts were treated with media, ECM, or CML-ECM (5 µg/mL) for 48 h, and cells were stained for TUNEL-positive cells. Representative images at 20× magnification with DAPI-stained nuclei (blue). Scale bar, 100 µm. Yellow arrowheads highlight tunnel-positive apoptotic cells. (**F**) Quantification of TUNEL-positive cells in total lung resident fibroblasts treated with media, ECM, or CML-ECM (5 µg/mL) for 48 h. One-way ANOVA was used (* *p* < 0.05, ** *p* < 0.001; n = 3/group).

To assess the effects of the ECM and CML-ECM in the survival of fibroblasts, we quantified the expression of anti-apoptotic BCL2 gene transcripts in fibroblasts treated with media, the ECM, and the CML-ECM. We observed a significant inhibition in the expression pro-survival Bcl2 gene transcripts by the ECM compared to media, and this decrease was attenuated with CML modification of the ECM (Figure 5A). We quantified changes in the protein levels of Bcl2 and Bcl-XL in lung resident fibroblasts treated with media, the ECM, and the CML-ECM for 72 h. Consistent with transcriptional changes, there was a significant decrease in Bcl2 and Bcl-XL protein levels in fibroblasts by the ECM compared to media, and this decrease was attenuated with CML modification of the ECM (Figure 5B–D). To determine the effects of the ECM and CML-ECM on the apoptotic clearance of fibroblasts, we treated lung resident fibroblast with media, the ECM, and the CML-ECM for 72 h and quantified TUNEL-positive fibroblasts. Consistent with changes in survival gene expression, we found a significant increase in the number of apoptotic cells treated with the ECM compared to media, and this increase was attenuated with CML modification of the ECM (Figure 5E,F).

## 3. Discussion

The findings of our study underscore the pivotal role played by interactions between the extracellular matrix (ECM) and fibroblasts in maintaining the structural integrity and functionality of lung tissue both in healthy states vis-a-vis during the course of pulmonary fibrosis. In this investigation, we have illuminated a novel and pathogenic role for AGEs, particularly carboxymethyllysine (CML) in the modification of ECM proteins. These CML modifications were found to trigger the aberrant activation of fibroblasts, which encompasses enhanced proliferation, fibroblast-to-myofibroblast transformation (FMT), prolonged cell survival or impaired clearance of fibroblasts, and excessive production of the ECM. Importantly, our immunostaining experiments have provided compelling evidence of the accumulation of CML adducts within fibrotic lesions and ECM extracts isolated from individuals afflicted with idiopathic pulmonary fibrosis (IPF), contrasting with the relatively lower levels detected in the lungs of both young and elderly individuals with healthy lung function. This observation strongly suggests a potential association between CML modification and the progression of fibrotic lung disease.

CML adducts, a subgroup of AGEs, arise as irreversible chemical modifications due to the non-enzymatic glycation of lysine residues on ECM proteins [14]. It is noteworthy that our study marks the pioneering effort to demonstrate that CML modifications may not only compromise the ECM’s intrinsic anti-fibrotic properties but also sustain and exacerbate pro-fibrotic activities in fibroblasts. Mechanistically, we have found that CML modification of the ECM contributes to improved fibroblast survival, which is partly attributable to an increased expression of anti-apoptotic proteins such as BCL2 and BCL-XL. Furthermore, our findings show an excessive production of collagen and FN1 by the CML-ECM in fibroblasts. Given that fibroblasts are the central cellular component of the ECM and play a fundamental role in collagen synthesis, the cumulative buildup of the CML-modified ECM may be accountable for perturbed ECM–fibroblast interactions, ultimately leading to excessive collagen production and tissue dysfunction. Consistent with our observations, several published studies have reported a positive correlation between AGE accumulation and the severity of fibrosis, particularly in diabetic nephropathy [27,32,33,34]. A recent published study suggests that the CML induces epithelial mesenchymal transformation in renal podocytes via transcription factor Zeb2, implicating its role in diabetic nephropathy [35]. Furthermore, the in vivo administration of an AGE cross-link breaker, phenyl-4,5-dimethylthiazolium bromide (ALT711), has been demonstrated to reduce myofibroblast formation in diabetic rats, which is concomitant with reduced levels of tubular CML adducts and TGFβ expression [36]. This provides further support for the underlying pathogenic connection between glycemia-induced AGE formation and myofibroblast activation. However, the formation of CML adducts in the lungs during aging compared to IPF is modest. Also, the effects observed with the CML-ECM in altering fibroblast activation are modest. This may suggest potential differences in CML adducts generated using in vitro methods compared in vivo. Therefore, it is important to determine the protein components of the ECM for their susceptibility to CML modification and altering fibroblast activity. Also, future studies are warranted to determine potential differences in the accumulation of CML adducts and other AGEs in IPF lungs compared to aging or hyperglycemia and measure differences in their effects on fibroblast activation.

To combat the adverse effects of AGEs on collagen production and fibroblast function, several therapeutic strategies have been explored. These encompass the development of AGE inhibitors, exemplified by aminoguanidine, and the modulation of receptor for AGEs (RAGE) signaling [30]. Furthermore, recent studies have shown that ellagic acid (EA) can mitigate bleomycin-induced pulmonary fibrosis by inhibiting the Wnt signaling pathway [37]. Additionally, lifestyle interventions, including dietary modifications and exercise regimens, have been proposed as potential means to reduce AGE accumulation [38,39]. Therefore, there exists a compelling need for a more rigorous assessment of fibroblast activation, considering CML adducts in conjunction with varying degrees of AGE accumulation in IPF. Given that AGE accumulation and fibroblast activation are gradual processes unfolding over several decades, it is anticipated that a meticulous examination of this phenomenon in human biopsies and through alternative preclinical models of pulmonary fibrosis such as aged mice with bleomycin injury and vascular damage will be imperative to conclusively determine the relevance of CML modification in the pathogenesis of pulmonary fibrosis. The inhibition of fibroblast activation holds immense clinical relevance in IPF, as it could potentially impede the progressive expansion of fibrotic lesions. Recent studies suggest that there are extensive post-translational modifications in tissues during oxidative stress, which could be a significant mechanism involved in fibrosis across various organs, including the lungs. These studies have highlighted the essential role of primary lung antioxidant defenses in animal models of pulmonary fibrosis and individuals with IPF. This includes key components such as catalase, glutathione (GSH), superoxide dismutase (SOD), and nuclear factor erythroid 2-related factor 2 (Nrf2), which play a protective role in the context of pulmonary fibrosis [40]. Another study provided evidence that extracellular superoxide dismutase (EC-SOD), an antioxidant enzyme that binds to syndecan, effectively hinders pulmonary fibrosis by suppressing inflammation and oxidative stress. This study also demonstrated that the absence of EC-SOD in the lung makes syndecan-1 susceptible to oxidative stress. Consequently, the shedding of the syndecan-1 ectodomain under oxidative conditions triggers neutrophil chemotaxis, hampers epithelial wound healing, and fosters fibrogenesis [41]. Nonetheless, the relationship between accumulation of the CML-modified ECM in inflammation and oxidative stress in the pathogenesis of pulmonary fibrosis remains incompletely defined, warranting further exploration.

**Figure 6:**
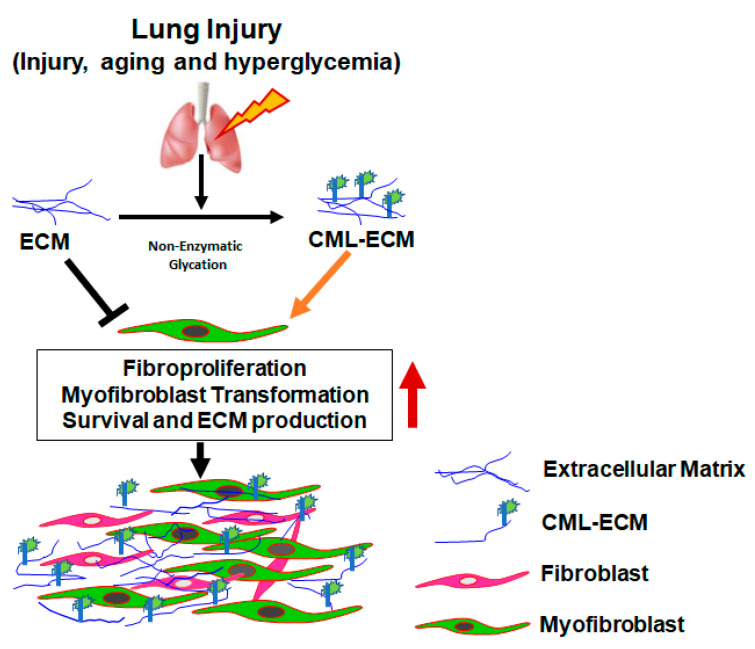
Schematic representation of CML-AGE formation in ECM during injury, aging and hyperglycemia. The accumulation of CML-ECM augments fibroblast activity including proliferation, myofibroblast transformation, survival and ECM production.

In conclusion, this study provides robust evidence for the accumulation of CML adducts within fibrotic lung lesions and their augmentation of fibroblast activation, thus offering a promising therapeutic target (Figure 6). Our findings underscore the possibility that CML-AGEs may play a pathogenic role in the development of IPF, particularly in the context of aging and/or hyperglycemia. This research not only deepens our understanding of the complex mechanisms underpinning pulmonary fibrosis but also opens up new avenues for future therapeutic interventions. The pro-fibrotic potential of the CML-modified ECM suggests that interventions aimed at inhibiting CML accumulation, by AGE inhibitors or AGE breakers, could provide scope to develop therapeutic for IPF. Hence, further studies are warranted to investigate potential anti-glycating agents that can either inhibit CML-AGE formation or modulate ECM–fibroblast interactions, thereby attenuating fibroblast activation in IPF and other fibrotic lung diseases.

## 4. Materials and Methods

### 4.1. Human Samples

IPF and healthy lung samples were obtained with the assistance of the Translational Pulmonary Science Center (TPSC), University of Cincinnati Medical Center. TPSC collects and maintains a repository of tissue and primary cells from individuals with chronic lung diseases. The local University of Cincinnati institutional review board (IRB #2013-8157) reviews the procedures in place to ensure adequate protection of human subjects and protection of patient privacy and confidentiality. The donor’s lung samples with no lung diseases were used as normal lung biopsies, and all materials were de-identified to the research team.

### 4.2. Extracellular Matrix (ECM) Proteins Extraction

Soluble ECM proteins were prepared as described previously with minor modifications [42]. The mouse and human lungs were perfused or rinsed thoroughly with decellularization buffer (8 mM CHAPS, 25 mM EDTA and 1 M NaCl in 1× PBS) and incubated at room temperature with rotation for 6–10 h. Then, tissue was washed with 1× PBS containing 10% penicillin/streptomycin 5–10 times for 5 min each to sterilize and remove the detergents. Tissue was further incubated with benzonase (90 U/mL) to digest all types of DNA and RNA in a buffer (50 mM Tris-HCl, 2 mM MgCl2 in 1× PBS, pH 7.4) for 30 min. To remove remnant DNA, the tissue was rinsed with PBS containing 10% FBS followed by homogenizing the lung extract in lysis buffer (50 mM Tris-HCl pH:7.4, 150 mM NaCl, 1% (*v*/*v*) Triton X-100, 0.5% (*w*/*v*) sodium deoxycholate + 1 mL SDS) containing freshly added proteinase inhibitors. Then, it was centrifuged at 10,000× *g* for 10 min, after which we collected the soluble ECM and dialyzed it extensively against 20 mM phosphate buffer to remove excess salts before using it to treat cells without and with CML modification. The presence of ECM proteins (e.g., collagen and fibronectin) was confirmed by immunoblotting analysis using antibodies specific to collagen and fibronectin. Proteins were quantified using BCA method.

### 4.3. Nε-Carboxymethyllysine Modification of ECM (CML-ECM)

The CML-ECM was prepared as described previously [21]. Briefly, ECM proteins (mouse or human, 1 mg/mL) were incubated in 1 mL of 0.2 M phosphate buffer, pH 7.8 containing glyoxylic acid (0.15 M) and NaBH3CN (0.45 M) for 24 h at 37 °C. After incubation, contents were subjected to extensive dialysis against the 20 mM phosphate buffer to remove the unbound glycating agents. Protein estimation was completed using the BCA method. CML modification was confirmed by immunoblotting using antibodies specific to CML. 

### 4.4. Histology and Immunohistochemistry

Immunostainings were performed as previously described [31]. Briefly, the lung tissues from IPF and healthy controls were fixed in 1:10 diluted formalin and embedded into paraffin blocks. The 5 µm thick sections were deparaffinized, after which antigen retrieval was performed using 10 mM citric acid (pH 6.0), blocked with 4% donkey serum, and incubated with primary antibodies followed by species-specific secondary antibodies. Hematoxylin counterstain was used to counterstain nuclei in blue color. DAB-stained sections were mounted and visualized using a Keyence BZ-X800 series microscope (Itasca, IL, USA) at 10× and 40× magnifications. BZ-X image analysis software version 1.31 was used to quantify brown staining areas in the total lung area of five representative images collected from each lung section and expressed as a percentage of the CML-AGE positive area in the total lung area of an image. The clinical parameters of normal and IPF patients are provided in Table 1. 

### 4.5. Preparation of Lung Resident Fibroblasts

Primary fibroblasts from the lungs of healthy human and mouse were isolated as described previously [43]. Briefly, lung pieces were finely minced with sterile razor blades and digested at 37 °C for 60 min in 5 mL of DMEM or IMDM containing collagenase type 3 (2 mg/mL; Cat: LS004182; Worthington Biochemical Corporation, Lakewood NJ, USA) for human or mouse lungs, respectively. Digested tissue was passed through a 100 µm filter, washed twice with media, plated onto 100 mm tissue-culture plates, and incubated at 37 °C, 5% CO2 to allow cells to adhere and migrate away from the larger remaining tissue pieces. To isolate lung resident fibroblasts, adherent lung cultures were harvested on days 5–8 with trypsin (0.05% trypsin, Life Technologies, Carlsbad, CA, USA), and cells were resuspended in a sterile buffer (0.5% FBS and 2 mM EDTA) containing 10 µL of anti-CD45 microbeads (Miltenyi Biotech, Auburn, CA, USA). To allow antibody binding, cells were incubated on ice for 15 min. Cells were then washed twice with sterile buffer and loaded onto magnetic columns (Miltenyi Biotech). Cells were then eluted with appropriate amounts of buffer in the presence of a magnetic field to isolate unbound lung resident fibroblasts (CD45- cells). Fibroblasts used in this study were between passage 1 and 3.

### 4.6. Immunoblotting

Immunoblot analysis was performed as described previously [44]. Briefly, total tissue lysates and primary cell lysates were prepared using the cell lysis buffer (Cell Signaling Technology, 9803S) containing protease inhibitors. A BCA protein estimation kit (Thermo Fisher Scientific, Waltham, MA, USA) was used to estimate the protein concentration. After SDS-PAGE separation, proteins were transferred to nitrocellulose membrane. The membranes were blocked with 5% BSA (MilliporeSigma, Burlington, MA, USA; A9647) for 2 h at room temperature and probed with specific primary antibodies including anti-CML (1:1000; Abcam, Boston, MA, USA, ab27684), anti-collagen1 (1:1000; Cell Signaling Technology, 91144S), anti-fibronectin1 (Santa Cruz Biotechnology, Dallas, TX, USA, Sc-9068, 1:500 dilutions), anti-smooth muscle actin alpha (1:20,000; Sigma-Aldrich, St. Louis, MO, USA), anti-Bcl2 (1:1000; Signaling Technology, 3498S), anti-Bcl-XL (1:1000; Cell Signaling Technology, 2764S) and anti-GAPDH (1:2000; Bethyl Labs, Montgomery, TX, USA, A300-641A) in blocking buffer at 4 °C overnight, which was followed by detection with HRP-linked appropriate secondary antibodies. The band intensities were quantified using Image Lab software version 6.1 (Bio-Rad, USA). The target proteins were normalized with internal control GAPDH.

### 4.7. Quantitative RT-PCR Analysis

The total RNA from lung tissues and primary cells was extracted using the RNeasy kit (Qiagen Sciences, Valences, CA, USA), and concentration was measured by a Nanodrop2000 spectrophotometer (Thermo Scientific, Madison, WI, USA) as previously described [45]. Reverse transcription was completed using SuperScript III (Thermo Fisher Scientific, Carlsbad, CA, USA), and real-time PCR was performed using SYBR select master mix (Bio-Rad) and a CFX384 Touch Real-time PCR instrument (Bio-Rad, Hercules, CA, USA) and analyzed using CFX maestro software version 4.0. Target gene transcripts from mouse samples were normalized to hypoxanthine–guanine phosphoribosyl transferase (Hprt) and human beta-actin for human transcripts. The RT-PCR primers (Invitrogen, Carlsbad, CA, USA, and IDT, Coralville, IA, USA) used in this study are listed in Supplementary Table S1.

### 4.8. BrdU Cell Proliferation Assay

Cell proliferation was assessed using the BrdU kit (Cell Signaling Technology, Denver, CO, USA), as previously outlined [31]. In brief, lung resident fibroblasts were subjected to treatment with media, the ECM, or the CML-ECM for 48 h in low-serum conditions (0.5% serum) with the addition of BrdU labeling solution after 24 h in culture. After 24 h of BrdU labeling, the cells were subsequently fixed, and BrdU immunodetection was performed following the manufacturer’s protocol.

### 4.9. TUNEL Assay

Fibroblasts were subjected to treatment with media, the ECM, or the CML-ECM for a duration of 48 h. Cells were fixed with 4% paraformaldehyde and nuclei were stained using DAPI, and the assessment of DNA fragmentation in apoptotic cells was carried out through the terminal deoxynucleotidyl transferase dUTP nick-end labeling (TUNEL) method, utilizing an In Situ Cell Death Detection kit, TMR red (Roche Diagnostics, Indianapolis, IN, USA). Staining procedures were executed in accordance with the manufacturer’s instructions. Imaging was performed at an original magnification of 20× using a Nikon AIR-A1 confocal microscope, and quantification was accomplished using MetaMorph imaging software (v6.2; Molecular Devices, San Jose, CA, USA).

### 4.10. Myofibroblast Transformation Assay

A fibroblasts-to-myofibroblast transformation assay was performed as described previously [31]. Lung resident fibroblasts isolated from the lung cultures of a MYHCreERT RosamTmG mouse model were treated with media, the ECM, or the CML-ECM along with 2 µM 4-hydroxy-tamoxifen (Sigma) for 72 h. Cells were then fixed with 4% paraformaldehyde, and nuclei were stained with DAPI. Fluorescence Images were obtained at 20× original magnification using a confocal microscope, and for the myofibroblasts quantification, the MetaMorph imaging software (v6.2; Molecular Devices) was employed.

### 4.11. Statistical Analysis

All data were analyzed using Prism (version 9.4.1 for Windows, GraphPad Software, San Diego, CA, USA). For multiple comparisons, 1-way ANOVA with Tukey’s test was performed. To determine statistical significance between 2 groups, Student’s 2-tailed *t*-test was used. All data were presented with mean ± SEM to indicate variability. *p* values of less than 0.05 were considered statistically significant.

## Figures and Tables

**Table 1:** Details of healthy controls and IPF patients.

Clinical Parameters	Normal		IPF
	Young	Old	
Age (years) Median (range)	25 (19–33)	58.5 (52–70)	62 (59–70)
Gender (male/female)	5/0	4/0	8/0
CML-AGE immunostaining (%)	1.4 ± 0.4	6.5 ± 0.5	19.9 ± 3.5

## Data Availability

No datasets analyzed or publicly archived related to this article.

## Supplementary Materials

The supporting information can be downloaded at https://www.mdpi.com/article/10.3390/ijms242115811/s1.
