# Supplementary material for: Nε-Carboxymethyl-Lysine Modification of Extracellular Matrix Proteins Augments Fibroblast Activation
Source: Int J Mol Sci. 2023 Oct 31;24(21):15811. doi: 10.3390/ijms242115811 (PMC10650592; doi:10.3390/ijms242115811)
Supplement: Supplementary file 1 [file ijms-24-15811-s001.zip › ijms-2639797-SI.pdf]

Supplementary data

N^{ε}-Carboxymethyl-lysine modification of extracellular matrix proteins augments fibroblast activation

Harshavardhana H. Ediga[1,2], Patrick Hester[1], Adithi Yepuri[1], Geereddy Bhanuprakash Reddy[2] and Satish K Madala[1*]

[1]Division of Pulmonary, Critical Care and Sleep Medicine, The University of Cincinnati, Cincinnati, OH, USA.

[2]Department of Biochemistry, ICMR-National Institute of Nutrition, Hyderabad, India.

***Corresponding Authors:** Dr. Satish K. Madala, Department of Internal Medicine, University of Cincinnati, 231 Albert Sabin Way, Cincinnati, OH 45267-0564, USA. Email: madalash@ucmail.uc.edu Phone: 513-558-1955 Fax: 513-558-4858.

Supplementary Table 1. The list of mouse and human RT-PCR primers used in the study.

Gene Symbol	Forward primer	Reverse primer
Mouse Acta2	TGACGCTGAAGTATCCGATAGA	CGAAGCTCGTTATAGAAAGAGTGG
Mouse AurkB	ATTGCAGACTTTGGCTGGTC	AATCATCTCTGGGGGCAGAT
Mouse Bcl2	TGTAGCTTTGCTCCCCTGAC	CACAGTGTTGGCAGGGTGT
Mouse Col1a1	CATGTTCAGCTTTGTGGACCT	GCAGCTGACTTCAGGGATGT
Mouse Fn1	CGGAGAGAGTGCCCCTACTA	CGATATTGGTGAATCGCAGA
Mouse Plk1	TTGTAGTTTTGGAGCTCTGTCG	CAGTGCCTTCCTCCTCTTGT
Mouse Hprt	GCCCTTGACTATAATGAGTACTTCAGG	TTCAACTTGCGCTCATCTTAGG
Human ACTA2	GCTTTCAGCTTCCCTGAACA	GGAGCTGCTTCACAGGATTC
Human AURKB	GATGGCCCAGAAGGAGAACT	AGGCTCTTTCCGGAGGACT

Human COL3A	CTGGACCCCAGGGTCTTC	CATCTGATCCAGGGTTTCCA
Human COL5A	CCTGGATGAGGAGGTGTTTG	CGGTGGTCCGAGACAAAG
Human β-ACTIN	CCAACCGCGAGAAGATGA	CCAGAGGCGTACAGGGATAG
